# Anthracycline-Related Heart Failure: Certain Knowledge and Open Questions: Where Do we Stand with Chemotherapyinduced Cardiotoxicity?

**DOI:** 10.1007/s11897-020-00489-5

**Published:** 2020-09-23

**Authors:** Emma Louise Robinson, Maral Azodi, Stephane Heymans, Ward Heggermont

**Affiliations:** 1 Cardiovascular Research Institute Maastricht, Maastricht University, Universiteitssingel 50, 6229 Maastricht, The Netherlands; 2 Luxembourg Centre for Systems Biomedicine, Université du Luxembourg, 6 Avenue du Swing, L-4367 Belvaux, Luxembourg; 3 Cardiovascular Centre Aalst, OLV Hospital Aalst, Moorselbaan 164, 9300 Aalst, Belgium

**Keywords:** Cardio-oncology, Anthracyclines, Cardiotoxicity, Chemotherapy-induced heart failure, Doxorubicin, Reactive oxygen species

## Abstract

In the last decade, cardio-oncology has become a discipline on its own, with tremendous research going on to unravel the mechanisms underpinning different manifestations of cardiotoxicity caused by anticancer drugs. Although this domain is much broader than the effect of chemotherapy alone, a lot of questions about anthracycline-induced cardiotoxicity remain unknown. In this invited review, we provide insights in molecular mechanisms behind anthracycline-induced cardiotoxicity and put it in a clinical framework emphasizing the need for patients to understand, detect, and treat this detrimental condition.

## Introduction

Anticancer therapy-associated cardiotoxicity, in particular chemotherapy-induced heart failure, is increasing as a clinical entity [1, 2]. Explanations for this phenomenon are an increased awareness of cardiotoxicity in patients receiving chemotherapy, a prolonged survival for young cancer patients exposing them to long-term cardiotoxicity risks, and an explosive growth of alternative anticancer drugs based on small molecules (e.g., tyrosine kinase inhibitors, proteasome inhibitors, etc.) [3, 4]. There is increased awareness that cardiotoxicity might even lead to premature morbidity and death in young cancer survivors. On the other hand, due to the fact that side effects of chemotherapy are often unpredictable, fear of cardiotoxicity sometimes leads to unnecessary and certainly inappropriate interruptions or restriction of potentially lifesaving cancer treatments. The different nature of the available anticancer drugs accounts for different cardiotoxicity risks, different times of onset, and subsequently different follow-up regimens. Moreover, it is also extremely challenging and hardly possible for the oncologist and cardiologist to be aware of all these specific side effects, sometimes even dependent on single molecule properties, not even class effects [5, 6].

Anthracycline-induced cardiotoxicity and subsequent heart failure is the best known clinical entity, especially in breast cancer patients and patients suffering from hematopoietic cancer such as lymphoma [7]. For anthracyclines, e.g., epirubicin and doxorubicin, cardiotoxicity is observed in up to 48% of patients following high-dose regimens, and on average, 1 in 5 anthracycline patients are affected [5]. However, these high doses are very rarely used—certainly in breast cancer—so the real-life risk of cardiotoxicity is lower than the aforementioned numbers, albeit that the risk of cardiotoxicity incrementally increases when different cardiotoxic products are combined [8, 9]. The difficulty of cardiotoxicity is that there are no specific risk factors nor predictors to adequately and timely assess cardiotoxicity risk in patients, and a late diagnosis confers a dismal prognosis, at least in anthracycline cardiotoxicity [10]. Furthermore, the detailed mechanisms behind this clinical entity vary dramatically and are as yet incompletely understood. In this review, we elaborate on what is known about anthracycline-related heart failure and we shed light on some interesting research avenues to pursue.

## Anthracycline-Related Heart Failure

### Past and Current Understandings

As early as 1967, about a decade after their use began, the first report for anthracycline-induced cardiotoxicity was observed in children receiving doxorubicin. The first thorough analysis of cases of anthracycline-associated cardiotoxicity (*in casu* adriamycin, or toxic cardiomyopathy) was described in 1973 by Lefrak and co-authors [11]. Since then, multiple reports of anthracycline-induced heart failure—cardiomyopathy—have been published. In the slipstream of research investigating the possible mechanisms behind cardiotoxicity, a classification into different types of cardiotoxicity was suggested in 2005. Type I cardiotoxicity (e.g., anthracycline cardiotoxicity) was related to myocardial apoptosis and necrosis in a dose-dependent manner, with a cumulative effect, causing permanent damage at the cellular level [12]. The reversibility of the disease is considered rather limited and the prognosis dismal. Type II cardiotoxicity (e.g., trastuzumab, bevacizumab) is related to interference with angiogenesis and is reversible if the causative therapy is interrupted [13]. However, there is now an overall consensus that this binary classification should be abolished. The detrimental effect of the so-called type II cardiotoxicity (exemplified with trastuzumab) was wrongfully underestimated because it was always compared with the irreversible damage caused by anthracyclines, whereas, in fact, with the new and emerging anticancer therapies, multiple toxicities do exist, shedding light on the ever more complex pathophysiology of anticancer therapy-induced cardiotoxicity.

### Diagnostic Challenges in Detecting Anthracycline Cardiotoxicity

There are two major concerns hampering effective detection of anthracycline-induced cardiotoxicity. The first is that the disease starts with subtle and subclinical changes that can hardly be detected by means of classical techniques, e.g., transthoracic echocardiography, although progress has been made in the field [14, 15]. The second problem is that anthracycline-related cardiotoxicity can occur much later, i.e., months or years after cessation of chemotherapy [5, 8].

**Fig. 1:**
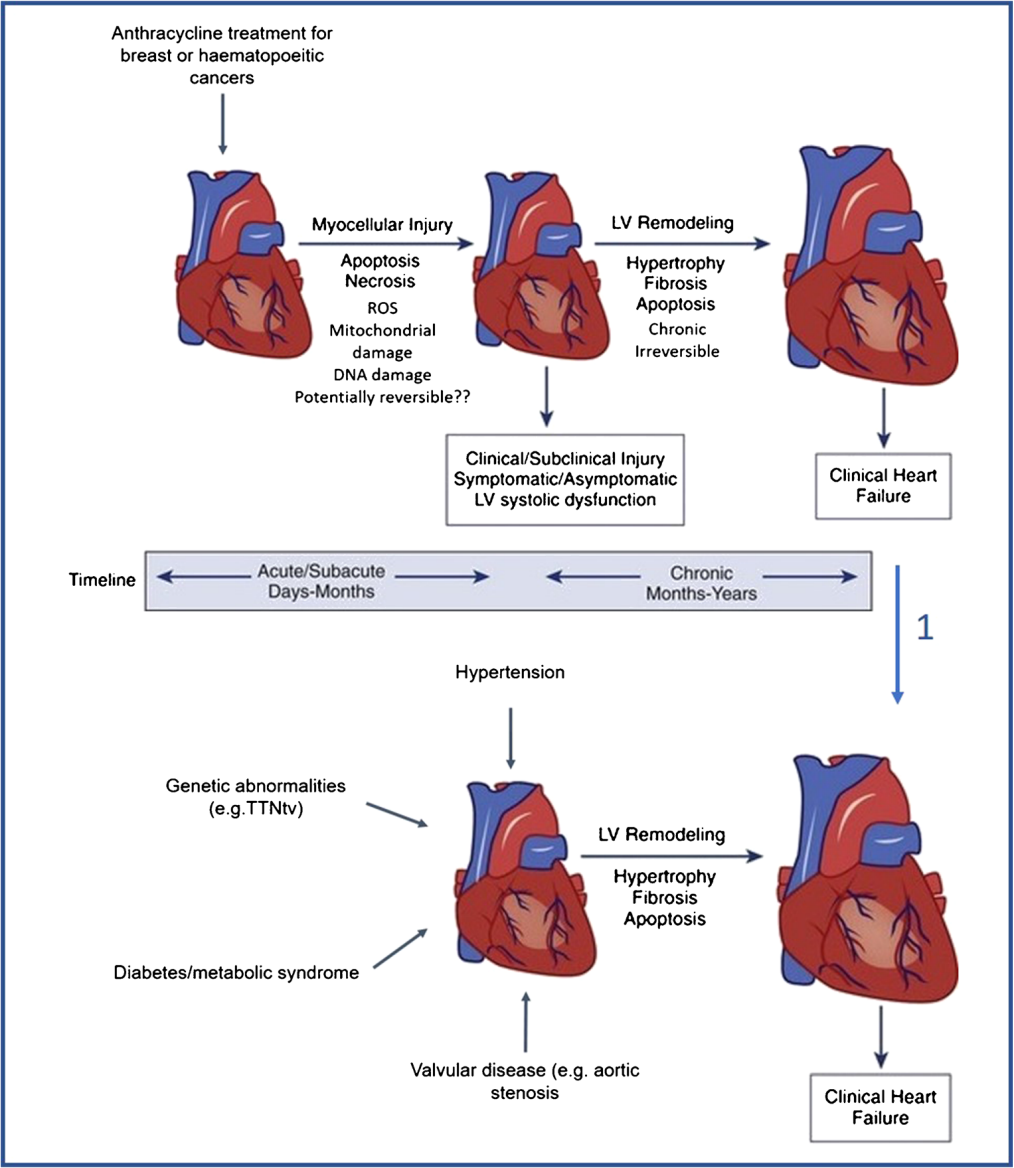
Central figure underpinning the potential mechanisms of chemotherapy-induced cardiomyopathy. Arrow 1: While it is currently impossible to distinguish a remodeled left ventricle, e.g., due to genetic abnormalities from a dysfunctional left ventricle due to chemotherapy, the mechanism is different. Arrow 2: We propose a two-staged mechanism for anthracycline-induced cardiomyopathy. In a first stage, there is myocellular injury (apoptosis, necrosis, ROS formation, etc), which is potentially reversible but might lead to subclinical injury, asymptomatic LV systolic dysfunction. In a second stage, these adverse signals give rise to subsequent LV remodeling with hypertrophy, fibrosis, and apoptosis, leading to a chronic irreversible chemotherapy-induced cardiomyopathy. While the first stage might only take days to months, the second chronic stage might last for years. ROS = reactive oxygen species, TTN = titin

Two distinct cardiotoxic effects of anthracyclines can be classified by the time of onset post cessation of treatment, as depicted in Fig. 1 [10]. Acute cardiotoxicity is apparent from immediately to within 1 year of treatment, whereas chronic late-onset cardiomyopathy can occur many years after cessation of the chemotherapy. Late-onset cardiotoxicity—often only discovered up to 15 years, sometimes longer, after treatment—is the one leading to chronic heart failure in cancer survivors and, therefore, represents a major clinical problem [16, 17].

The present understanding of the mechanisms behind the cardiotoxic effects of neoplastic treatment is remarkably limited. The suitability of classic circulating diagnostic biomarkers for heart failure is inadequate. Prospective tools to identify patients that will develop acute and delayed cardiomyopathy following anticancer therapy are currently nonexistent. Early diagnostic of the patients prone or subjected to develop an (acute or late) anthracycline-induced cardiotoxic event would allow adapting the cancer treatment immediately and managing cardiotoxicity with cardioprotective drugs.

The in-depth assessment of diagnostic challenges is however beyond the scope of this review. A detailed flowchart on the diagnosis of anthracycline-related cardiotoxicity is available in the position paper of the European Society of Cardiology, 2016 [5].

### Prevention of Cardiotoxicity: Dexrazoxane

Given the reasonably irreversible nature of late-onset anthracycline-induced cardiotoxicity, a strategy aiming at preventing the disease is paramount. Randomized trials have been performed to explore primary prevention in patients undergoing anthracycline chemotherapy. One of the best studied molecules is dexrazoxane. This molecule, which is a derivative of ethylenediaminetetraacetic acid (EDTA), has been licensed in several countries for the prevention of cardiotoxicity due to anthracycline-based chemotherapy as well as for the treatment of accidental extravasation of anthracyclines. A thorough review on different aspects of this molecule has been published in the past [18]. In general, it can be stated that indeed, dexrazoxane prevents—at least in part—cardiotoxicity caused by anthracyclines. Furthermore, in large meta-analyses, the administration of dexrazoxane does not significantly reduce overall survival or progression-free survival from the cancer for which the anthracyclines were initially administered [19]. Other options to prevent anthracycline-induced cardiotoxicity are the administration of a liposomal form or a less toxic form of anthracycline [20–23]. Prevention of cardiotoxicity is nevertheless much broader than the administration of dexrazoxane, which is grossly only effective in anthracycline-related cardiotoxicity. Numerous trials with classical heart failure therapies (beta blockers, angiotensin-converting enzyme (ACE) inhibitors, ARBs) have been undertaken to prevent cardiotoxicity. However, until recently, no unequivocal clinical benefit of these molecules was observed, except maybe for patients with asymptomatic troponin elevation [24].

### Treatment Options for Anthracycline-Induced Cardiotoxicity

So far, no clear guidance exists on the treatment of patients with symptomatic heart failure due to anthracycline-induced cardiotoxicity. In the detailed position paper of the European Society of Cardiology, it is advised that patients be treated according to the most recent guidelines for the treatment of heart failure [5, 25]. In circumstances of severe instability, chemotherapy should also be interrupted. In the case of a recuperated LV function and a rechallenge with the same or similar chemotherapy, the initiation or continuation of ACE inhibitors and beta blockers is recommended.

## Molecular Basis of Anthracycline-Related Heart Failure

### Overview

The molecular mechanisms for cardiotoxicity induced by anthracyclines include disruption of transcription by topoisomerase II, mitochondrial iron accumulation, oxidative stress such as lipid peroxidation and protein nitrosylation, mitochondrial dysfunction, and Ca^2+^ handling abnormality [26]. Generation of reactive oxygen species (ROS) is considered the most significant mechanism of chemotherapy-induced cardiotoxicity. One of the mechanisms of action for doxorubicin-induced cardiotoxicity is related to the excessive generation of ROS followed by oxidative stress [27]. Thus, it is obligatory to keep the proper level of ROS because of its significant role in hemostasis, cell proliferation, and cell death and its direct effects on DNA, RNA, proteins, and lipids [28]. Mitochondrial permeability transition, outer membrane rupture, release of apoptotic signaling molecules, and irreversible injury to the mitochondria are the other implications of excess ROS production [29]. Although the mechanism of action of anthracycline cardiotoxicity is not completely clarified, the most widely accepted hypothesis for the mechanisms of anthracycline-induced cardiotoxicity is mediated by topoisomerase IIβ (Top2b) specifically in cardiomyocytes. Deletion of Top2b protects cardiomyocytes from doxorubicin-induced DNA double-strand modifications [30].

### Crucial Role for ROS Formation in Mitochondria

In anticancer therapy, the mitochondria are a preferential place for doxorubicin-induced ROS overproduction mediated by the activity of mitochondrial NADPH oxidase (mitoNOX). Essentially, nitric oxide synthase (NOS) converts l-arginine to nitric oxide (NO) in the presence of molecular oxygen while using NADPH as reductant. Under the circumstance of lower levels of l-arginine or cofactor (6R)-5,6,7,8-tetrahydrobiopterin (BH4), formation of superoxide instead of NO may occur [31]. Vásquez-Vivar et al. have shown that doxorubicin binds to endothelial NOS, increasing superoxide formation and reducing nitric oxide production [32]. In the oxidoreductive reaction, a single electron is transferred from NADPH to doxorubicin. This forms a semiquinone radical which, when complexed with iron, is responsible for oxygen reduction, producing a superoxide ion. In addition, the interaction between doxorubicin and cardiolipin forms a drug–phospholipid complex that, in turn, can inhibit mitochondrial enzymes involved in oxidative phosphorylation. Mitochondrial membrane damage can also cause the inactivation of sodium and calcium transporters. These transporters are involved in ion homeostasis. Cardiolipin is rich in polyunsaturated fatty acids, which is located in the inner mitochondrial membrane and is required for the activity of the respiratory chain [33]. Cardiac tissue is rich in mitochondria and its cytotoxic potential of anthracyclines may be magnified by a high density of mitochondria and lower levels of enzymatic capacity to detoxify ROS [34].

## Animal and Cellular Models of Anthracycline-Related Heart Failure

### Animal Models of Anthracycline Cardiotoxicity

Mouse, rat, rabbit, pig, and dog are the common experimental models for in vivo studies. The advantage of using animal studies is due to being able to perform repeated administration of chemotherapeutic agents, thereby mimicking chronic cardiotoxicity in clinical practice [35]. A comprehensive overview of in vivo experimental studies into the cardiotoxicity of anthracyclines, along with the advantages and disadvantages of each experimental model, is presented in Table 1. Rabbits were the first long-term animal for anthracycline-induced cardiotoxicity by demonstrating myocardial damage and fibrosis [44]. Richard et al. showed that doxorubicin-treated rats have increased oxidative stress and pathological remodeling with lower left ventricular contractility, higher levels of thiobarbituric acid or dihydroethidium fluorescence—which are plasma and myocardial oxidative stress markers—and markedly altered transcript levels for all measured markers of cardiac remodeling, except VEGF-A [41]. Expression of inducible NOS at the RNA and protein levels was significantly increased in male Wistar rats following 8 weeks of adriamycin treatment, which continued to increase up to 10 weeks of treatment [42]. Also, human-induced pluripotent stem cell (iPSC)-derived cardiomyocytes generated from patients with depressed left ventricular ejection fraction (LVEF) demonstrate increased cell death and ROS production following in vitro doxorubicin treatment [45].

**Table 1:** Overview of doxorubicin-induced toxic effects in animal models

Animal models	Mechanism of action	
Mice	Inhibit with topoisomerase IIβ and induce apoptosis [30]	Advantages: • Low purchase and maintenance costs • More suitable for “high-throughput” studies than large animal models • Up to 99% genetic similarity between human and murine genes • Relatively short breeding time and high breeding rate—suitable for generation of genetically modified mouse lines • Short gestation time (21 days) • Ideal for rapid establishment of proof-of-principle, discovery, and functional data, which can then be applied to other experimental models and, eventually, into humans Disadvantages: • Typically do not recapitulate all aspects of human cardiovascular disease (e.g., mice rarely develop atherosclerosis of the coronary arteries) • Murine cardiac physiology poorly replicates that of humans, including cellular electrophysiology and Ca^2+^ transport and predominant myosin heavy chain (MHC) α expression in the adult heart over β-MHC, as compared with other small animal models mentioned here • They are phylogenetically far from humans and some pathophysiological features of disease and their response may not be reliable predictors • Translational aspects and the value of genetic mouse models must be interpreted with caution • Mice have a differential response than humans to anthracyclines, an exacerbated acute cardiac response. Protocols for anthracycline treatment in mice that mimic the human phenotypic response are yet to be established
Mice	Increased fibrosis, cardiomyocyte diameter, and apoptosis [36]	
Mice	Dual involvement of apoptosis and necrosis Doxorubicin-induced cardiomyopathy is dependent on BAX [37]	
Mice	NADPH oxidase (NOX) plays an important role in the progress of the oxidative signal transduction and DOX-induced cardiomyopathy [38]	
Mice	The effects of myeloid differentiation protein 1 (MD-1) in pathological cardiac remodeling and myocardial ischemia/reperfusion (I/R) injury [39]	
Mice	Reduction of oxidative stress and cardiomyocyte apoptosis in DOX-induced cardiotoxicity via maintaining AMPKα/UCP2 pathway [40]	
Rats	The upregulation of inducible NOS (iNOS) gene and protein [41]	Advantages: • Low purchase and maintenance costs • Easy to handle—some strains more docile than mice and experimental interventions easier on a slightly larger animal than mice • Short gestation period (21–23 days), high breeding rate, and litters of up to 15 pups • Rats treated with adriamycin are usually used to investigate the mechanisms of cardiotoxicity and ways of preventing it Disadvantages: • Few genetically modified rat lines, making experimental cardiology using genetic interventions difficult • Not useful in investigating mechanisms of stroke
Rats	A decrease in calcium-loading capacity together with alterations in cardiac mitochondrial function has been observed [42]	
Rabbits	Leads to Ca^2+^ overload [43]	Advantages: • Rabbits have similar cardiomyocyte cellular electrophysiology properties and Ca^2+^ transport system to humans or larger animals (e.g., dogs and pigs) as well as predominant expression of myosin β-MHC over α-MHC, as for humans • Most studies using rabbit models of anthracycline-induced cardiomyopathy have concentrated on the evaluation of potential cardioprotective agents • Can develop atherosclerosis with high fat feeding, for example Disadvantages: • Higher maintenance costs than rats and mice • Harder to handle • Few transgenic rabbit lines. Genetic intervention for functional experiments uncommon in rabbits
Rabbits	Myocardial damage and fibrosis [44]	

Juvenile mice were treated with doxorubicin for a period of 5 weeks (25 mg/kg cumulative dose) from 2 weeks of age. A reduction of cardiac systolic function due to atrophy of the heart, low levels of cardiomyocyte apoptosis, and decreased growth velocity were observed. However, most adult murine models still require acute drug administration which might skew the observations [46]. Rea et al. generated a mouse model of cardiotoxicity induced by doxorubicin showing early remodeling of the left ventricle. In addition, histological analysis revealed increased fibrosis, increased cardiomyocyte diameter, and apoptosis [36].

Overexpression of eIF5A induced by doxorubicin in H9c2 cardiomyocyte leads to a gradual increase in ROS generation, growth perturbation, and initiation of apoptosis. Mitochondrial dysfunction becomes prominent after a gradual increase in ROS generation. In addition, increased Ca^2+^ influx in the mitochondria leads to loss of the mitochondrial transmembrane potential, release of cytochrome *c*, and caspase activation [47]. Intrinsic apoptosis pathway or mitochondrial apoptosis pathway is initiated by intracellular stress such as oxidative stress, calcium overload, and DNA damage, leading to Bax/Bak-dependent mitochondrial outer membrane permeabilization (MOMP) and release of cytochrome *c* from the mitochondria into the cytosol. Cytosolic cytochrome *c* and apoptotic protease-activating factor 1 (Apaf-1) result in activation of caspase 9 [48].

Due to the time, costs, and differential long-term effects of anthracycline treatment in small experimental animal models, studies into the late-onset cardiotoxic effects and, therefore, understanding of underlying molecular mechanisms are lacking (Fig. 1).

### Cell Culture Models of Anthracycline Cardiotoxicity

In in vitro cell culture models, including isolated cardiac myocytes (particularly primary neonatal rat cardiomyocytes or less often adult cardiomyocytes), cardiomyocyte-derived cell lines (e.g., H9c2 rat embryonic cardiomyoblasts) are the most straightforward approach due to their ease of use to investigate gene function in a high-throughput manner and lower cost [49]. Merten et al. reported doxorubicin as a potent inducer of apoptosis in H9c2 cardiomyocytes by activation of PI3K/Akt and, to a lesser extent, calcineurin [50]. MicroRNA-21 expression significantly increases in both mouse heart tissue and H9C2 cells after treatment by doxorubicin. The mechanism of action for this microRNA is related to the modulation of the antiproliferative factor, B cell translocation gene 2 [51].

Tumor necrosis factor-α (TNF-α), Fas ligand (FasL), and TNF-related apoptosis-inducing ligand (TRAIL) are extracellular stress signals. By binding to their individual death receptors—TNF-α receptor 1 (TNFR1), Fas, and TRAIL receptor 1/2 (TRAILR1/2), extrinsic apoptosis will take place. Fas-associated death domain (FADD) is recruited by death receptors. Finally, caspase activation causes apoptotic death of the cell [48]. Doxorubicin induces cardiotoxicity through upregulation of death receptors (DRs) (TNFR1, Fas, DR4, and DR5) in iPSC-derived cardiomyocytes at both the protein and mRNA levels. Spontaneous apoptosis is enhanced by physiologically relevant death ligands including TRAIL. Induced death receptors in cardiomyocytes could be a critical mechanism by which doxorubicin causes cardiotoxicity [52].

Increased MMP1, IL-6, TGF-β, and collagen expression in human cardiac fibroblasts (HCFs) is followed by doxorubicin treatment. This suggests a new—or additive—mechanism of doxorubicin-induced cardiotoxicity [53]. Treatment of the murine HL-1 cardiomyocyte cell line or isolated adult rat ventricular myocytes with doxorubicin decreased GATA-4 protein and mRNA levels. Prevention of GATA-4 DNA-binding activity by adenoviral-mediated expression of dominant negative mutant of GATA-4 induced apoptosis. Downregulation of GATA-4 inciting cardiomyocyte apoptosis could be a mechanism of anthracycline-induced cardiotoxicity [54]. A complete reference table of in vitro functional experiments can be found in Table 2.

**Table 2:** Overview of doxorubicin-induced toxic effects in cell lines

Cell line	Mechanism of action
H9c2	Induces the release of apoptosis-inducing factor (AIF) from the mitochondria [55]
H9C2	*Fndc5* deficiency resulted in increased oxidative damage and apoptosis [56]
H9c2	Apoptosis due to the accumulation of eIF5A [47]
H9c2	Involved the activation of PI3K/Akt and the PI3K–Akt signaling pathway seems to be critically involved in DOX-induced hypertrophy [50]
H9c2	Induced senescence by marked increases in the expression of p53 and p16 [57]
hiPSC-CMs	Apoptotic and necrotic cell death, ROS production, mitochondrial dysfunction, and increased intracellular calcium concentration and decreased antioxidant pathway activity [58, 59]
hiPSC-CMs	Increased expression of p53 and DR. DR expression might function as a predictive biomarker for cardiac damage [60]
iPS-CMs	Upregulated the expression of death receptors (DRs) (TNFR1, Fas, DR4, and DR5) [61]
HL-1	Downregulation of GATA-4 and the induction of apoptosis [54]
HCF	Increased MMP1, IL-6, TGF-β, and collagen expression promoted Akt and Smad phosphorylation [53]
Neonatal rat ventricular myocytes (NRVMs)	Doxorubicin activated CaMKII and NF-κB through their phosphorylation and increased cleaved caspase 3 in cardiomyocytes [62]
H9c2 and mouse embryonic fibroblasts (MEFs)	Proteosome inhibitor (bortezomib and MG-132) administration prevented doxorubicin-induced topoisomerase IIβ-mediated DNA damage. Topo IIβ knockout in MEFs phenocopied proteasome inhibitor treatment [63]

## Conclusions

Cardiotoxicity in general and anthracycline-induced heart failure in particular are fast evolving scientific research avenues. Despite increasing knowledge on the mechanisms of cardiotoxicity, timely diagnosis of subclinical cardiac dysfunction remains a difficult item. Furthermore, there is no curative treatment for the disease—except for general heart failure medications. Further mechanistic research is warranted in order to discover a disease-specific treatment.
